# Post-Transcriptional Regulation of the MiaA Prenyl Transferase by CsrA and the Small RNA CsrB in *Escherichia coli*

**DOI:** 10.3390/ijms26136068

**Published:** 2025-06-24

**Authors:** Joseph I. Aubee, Kinlyn Williams, Alexandria Adigun, Olufolakemi Olusanya, Jalisa Nurse, Karl M. Thompson

**Affiliations:** 1 Department of Microbiology, College of Medicine, Howard University, Washington, DC 20059, USA; 2 Department of Biology, Claflin University, Orangeburg, SC 29115, USA; 3 Department of Biology, Howard University, Washington, DC 20059, USA

**Keywords:** small RNA, tRNA modification, RNA binding protein, RNA processing

## Abstract

MiaA is responsible for the addition of the isopentyl modification to adenine 37 in the anticodon stem loop of specific tRNAs in *Escherichia coli*. Mutants in *miaA* have pleotropic effects on the cell in *E. coli* and play a role in virulence gene regulation. In addition, MiaA is necessary for stress response gene expression by promoting efficient decoding of UUX-leucine codons, and genes with elevated UUX-leucine codons may be a regulatory target for i^6^A-modified tRNAs. Understanding the temporal nature of the i^6^A modification status of tRNAs would help us determine the regulatory potential of MiaA and its potential interplay with leucine codon frequency. In this work, we set out to uncover additional information about the synthesis of the MiaA. MiaA synthesis is primarily driven at the transcriptional level from multiple promoters in a complex operon. However, very little is known about the post-transcriptional regulation of MiaA, including the role of sRNAs in its synthesis. To determine the role of small RNAs (sRNAs) in the regulation of *miaA*, we constructed a chromosomal *miaA*-*lacZ* translational fusion driven by the arabinose-responsive P_BAD_ promoter and used it to screen against an *Escherichia coli* sRNA library (containing sRNAs driven by the IPTG-inducible P*_Lac_* promoter). Our genetic screen and quantitative β-galactosidase assays identified CsrB and its cognate protein CsrA as potential regulators of *miaA* expression in *E. coli*. Consistent with our hypothesis that CsrA regulates *miaA* post-transcriptional gene expression through binding to the *miaA* mRNA 5′ UTR, and CsrB binds and regulates *miaA* post-transcriptional gene expression through sequestration of CsrA levels, a deletion of *csrA* significantly reduced expression of the reporter fusion as well as reducing *miaA* mRNA levels. These results suggest that under conditions where CsrA is inhibited, *miaA* mRNA translation and thus MiaA-dependent tRNA modification may be limited.

## 1. Introduction

MiaA is a tRNA Isopentenyl Transferase (IPT or IPTase) that catalyzes the prenylation of adenine 37 in the anticodon stem loop of tRNAs that read codons beginning with uridine [1,2,3]. The resulting N^6^-(isopentenyl) adenosine 37 (i^6^A37) is the precursor for subsequent methylthiolation by the MiaB enzyme, resulting in the 2-methylthio-N^6^-(isopentenyl) adenosine (ms^2^i^6^A37) in *E. coli* [1,2,3]. The function of MiaA has been the subject of study for several decades [1,2,3,4,5,6,7]. Previous studies demonstrated a role for i^6^A37, and other RNA modifications, in translational fidelity by preventing translational aberrations such as ribosome pausing and ribosomal frameshifting [8,9,10,11,12,13,14,15]. In addition, *E. coli miaA* mutants decrease cellular growth rates and promote spontaneous mutants, specifically GC—TA transversions [2,3,16]. The MiaA amino acid sequence is highly conserved with homologues in both prokaryotes and eukaryotes [17,18,19]. MiaA levels influence translational frameshifting to alter the global proteome, fitness, and virulence potential of Extraintestinal Pathogenic *E. coli* (ExPEC) [20]. MiaA promotes virulence in *Shigella flexneri* [21]. *Acinetobacter baumannii miaA* mutations exhibit Colistin resistance [22]. *Streptomyces albus* requires *miaA* for proper morphological development and metabolic regulation [23,24].

We previously identified MiaA as a regulatory factor necessary for the full expression of the stationary phase and general stress response sigma factor RpoS in *E. coli* K12 (σ^S^) [25]. MiaA affects the expression of two additional stress response genes in *E. coli* K12: Hfq and IraP, which are also involved in the regulation of RpoS [26,27]. The MiaA (i^6^A)-sensitive genes identified in *E. coli* K12 thus far have higher UUX-leucine codon usage than the average genome wide UUX-leucine codon usage [25,27]. MiaA promotes the expression of its targets by promoting efficient UUX-leucine decoding [27]. While these studies have expanded our understanding of i^6^A modification function, we still do not know the physiological or metabolic conditions that control i^6^A levels in *E. coli*. We reasoned that further characterization of MiaA synthesis would assist us in understanding the regulation of i^6^A levels in the cell and we sought to achieve that in this work.

The *miaA* gene is contained in a complex operon, immediately upstream of *hfq*, the gene encoding the global RNA chaperone that also serves as a host factor for bacteriophage Qβ replication [28,29,30]. The transcription of *miaA* is driven by two promoters, one of which is a heat shock promoter (*miaA*_P2(hs)_) that is recognized by the heat shock-responsive alternative sigma factor, σ^32^ [30,31]. However, we know much less about the post-transcriptional regulatory factors that influence *miaA* expression. There are several clues in the literature that point to potential post-transcriptional regulation of MiaA. First, the transcript driven by the *miaA*_P2(h)_ promoter has a 270 nucleotide 5′ untranslated region (UTR) [30,31]. Long 5′ UTRs are often associated with post-transcriptional regulatory processes. Second, a null mutation in *hfq* resulted in elevated levels of multiple transcripts from the *miaA* superoperon [32]. Third, MiaA transcript levels are increased in the absence of RNase E and/or RNaseIII [31]. RNase E is an endoribonuclease that is essential for growth and works with the 3′ to 5′ exoribonuclease polynucleotide phosphorylase (PNPase) to process rRNA and tRNA and the coordinated turnover of sRNAs and their mRNA targets [33,34,35,36]. These previous results support the idea that MiaA expression is regulated at the post-transcriptional level. Post-transcriptional regulation of *miaA* by Hfq and RNase would likely be mediated by sRNAs. Yet, prior to this work, no sRNA regulators of *miaA* have been identified. To close this gap, we executed a targeted screen of a plasmid sRNA library on a P*_BAD_*-*miaA27_P2_*-*lacZ* translational fusion strain, as previously described [37,38,39]. We identified several candidate sRNA repressors of the *miaA*_P2(hs)_ transcript including SdsR, ArcZ, GcvB, Spot42, and CsrB. While SdsR and CsrB had the most dramatic inhibitory effect of all candidate sRNAs regulators of the *miaA*_P2(hs)_ transcript in our genetic screen, the effect of CsrB on *miaA* expression was the focus of subsequent experiments for this study.

CsrB is an sRNA that acts to sequester the activity of the RNA-binding protein CsrA [40]. CsrA is a pleiotropic regulator of carbon metabolism and a global RNA-binding protein involved in direct post-transcriptional regulation of gene expression in *E. coli*, following binding to the 5′ untranslated regions [41,42,43]. CsrA regulates the expression of *pgaABCD* and *flhDC* operons to influence biofilm formation and motility/flagellar synthesis, respectively, in *E. coli* [44,45]. CsrA binds to several mRNA transcripts and subsequently regulates the expression of these genes in *E. coli*. The regulatory processes, metabolic impact, and virulence-promoting activities of CsrA have been identified in many other bacteria including *Escherichia coli*, *Salmonella Typhimurium*, *Pseudomonas* sp, *Serratia* sp., *Campylobacter jejuni*, *Vibrio cholera*, *Yersinia pseudotuberculosis*, *Erwinia amylovora*, *Legionella pneumophila*, *Bacillus subtilis*, *Staphylococcus aureus*, *Clostridiodes difficile*, and *Acinetobacter baumannii* [46,47,48,49,50,51,52,53,54,55,56,57,58,59,60,61]. CsrA activity was previously demonstrated to be regulated through sequestration by the sRNAs CsrB and CsrC. However, recent studies have demonstrated an expanded number of 10 direct CsrA sRNA-binding partners in vitro [62]. Four of them bind to CsrA in vivo [62]. In addition, recent studies suggest that CsrA binds sRNAs and promotes sRNA–mRNA complex formation, placing it in the category of RNA chaperones such as Hfq and ProQ [57,63,64]. Since CsrA works with CsrB to regulate gene expression, we tested the effect of CsrA on MiaA expression. We show that CsrA is necessary for *miaA*_P2(hs)_ translation. We further extended the prior work on RNases and MiaA expression, demonstrating that RNase E and PNPase from the Degradosome contribute to stabilization of the *miaA* mRNA transcript.

## 2. Results

### 2.1. CsrB Was Selected as a Multi-Copy Repressor of MiaA Translation in a Targeted Screen of sRNA Regulators

**Figure 1:**
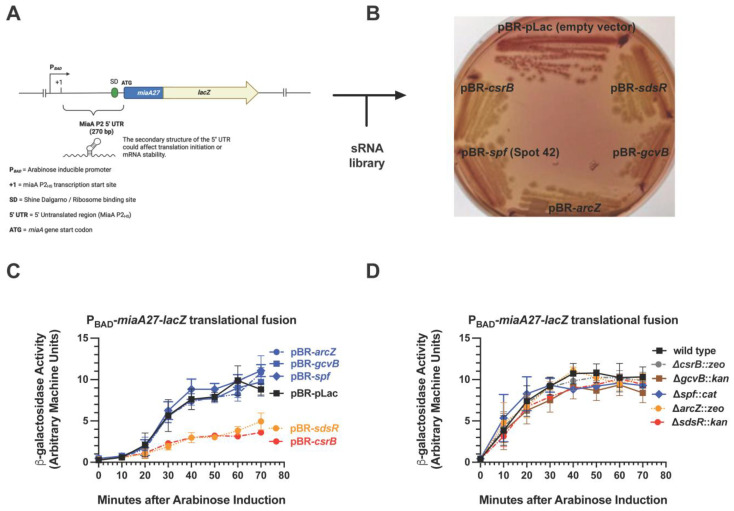
Small RNA library screen for regulators of *miaA* expression. (**A**) A schematic representing the arabinose-inducible *miaA*-*lacZ* translational gene fusion (P*_BAD_*-*miaA27*_(P2HS)_-*lacZ*) containing the 5′ UTR from the *miaA* P2 heat shock promoter. (**B**) A P*_BAD_*-*miaA27*_(P2HS)_-*lacZ* translational fusion strain was transformed with a library of 30 known sRNAs cloned downstream from an IPTG-inducible promoter in plasmid pBR-pLac and screened for activity on MacConkey-Lactose plates supplemented with ampicillin. Results shown are for sRNA clones that gave a Lac- phenotype, suggesting a role for these sRNAs in the negative regulation of MiaA expression. (**C**) Quantitative β-galactosidase assay analysis of P*_BAD_*-*miaA27*_(P2HS)_-*lacZ* translational fusion strain (JIA4000) carrying pBR-pLac (empty vector—JIA4001), pBR-*sdsR* (JIA4010), pBR-*arcZ* (JIA4018), pBR-*gcvB* (JIA4015), pBR-*spf* (JIA4024), or pBR-*csrB* (JIA4029). Each time point represents an average of at least three experiments and error bars represent the standard error of the mean (mean ± sem). (**D**) Quantitative β-galactosidase assay analysis of P*_BAD_*-*miaA27*_(P2HS)_-*lacZ* translational fusion strain (JIA4000) with deletions–insertions in the genes for the candidate sRNA repressors of *miaA* picked up in our screen: Δ*csrB*::*zeo* (JIA4042), Δ*gcvB*::*kan* (JIA4041), Δ*spf*::*cat* (JIA4043), Δ*arcZ*::*zeo* (JIA4040), and Δ*sdsR*::*kan* (JIA4045). Each time point represents an average of at least three experiments and error bars represent the standard error of the mean (mean ± sem). β-galactosidase activity is quantified using arbitrary machine units as described in Section 4.

**Figure 2:**
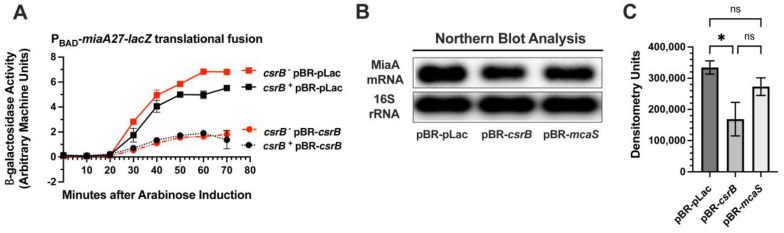
The effect of CsrB on *miaA* expression. (**A**) Quantitative β-galactosidase assay analysis of P*_BAD_*-*miaA27*_(P2HS)_-*lacZ* translational fusion activity showing repression by CsrB sRNA. β-galactosidase assays were repeated at least three times and data points represent the mean plus and minus the standard error of the mean (mean ± sem). β-galactosidase activity is quantified by the use of arbitrary machine units as described in Section 4. (**B**) Northern blot analysis of MiaA steady-state levels following over-expression of CsrB (KMT792) or McaS (KMT799) in comparison to the empty vector. Northern blots were repeated at least three times. (**C**) Quantitative densitometry of Northern blot analysis. Densitometry signals were acquired using a Fluorochem R Fluorescent/Chemiluminescent imager. Each data point represents an average of at least three experiments and error bars represent the standard error of the mean (mean ± sem) based on the densitometry of Northern blot signals in Section (**B**). Statistical analysis was executed using One-Way ANOVA with Tukey’s Multiple Comparisons test on GraphPad Prism 9 (* *p*-value = 0.05, ns = not significant).

We constructed a chromosomal *miaA*-*lacZ* translational fusion, whose expression is driven by the arabinose-responsive P*_BAD_* promoter (Figure 1A). It was used for screening a plasmid-based sRNA library containing 30 *Escherichia coli* sRNAs that were cloned downstream of an IPTG-inducible promoter (Mandin and Gottesman, 2009 [38]). The screening was carried out on Mac-Lac-Amp plates supplemented with arabinose to a final concentration of 0.002% to induce basal transcription from the P*_BAD_* promoter without producing a strong Lac^+^ phenotype, as previously described [65]. This facilitated the identification of sRNA repressors more easily through our genetic screen. We identified five candidate sRNA regulators of *miaA*: SdsR, ArcZ, GcvB, Spot42, and CsrB, with CsrB showing the strongest inhibitory effect (Figure 1B). To further test our hypothesis that post-transcriptional regulation of *miaA* is modulated by one or more sRNAs, we executed quantitative β-galactosidase assays of the P*_BAD_*-*miaA*-*lacZ* translational fusion strains carrying plasmids expressing SdsR, ArcZ, GcvB, Spot42, or CsrB (Figure 1C). Over-expression of ArcZ, GcvB, and Spot42 did not affect P*_BAD_*-*miaA*-*lacZ* activity as compared to the vector control (Figure 1C). There was a 2-fold decrease in the activity of P*_BAD_*-*miaA*-*lacZ* translational fusion activity upon over-expression of CsrB or SdsR (Figure 1C), suggesting that CsrB and SdsR sRNAs are involved in the post-transcriptional repression of *miaA* expression. We also tested the impact of deleting the sRNA candidates on the expression of the P*_BAD_*-*miaA*-*lacZ* translational fusion (Figure 1D). Individual deletions of *gcvB*, *spf*, *arcZ*, or *sdsR* had no effect on the expression of the activity of the P*_BAD_*-*miaA*-*lacZ* translational fusion following arabinose induction (Figure 1D). The *csrB* mutant demonstrated a modest increase in activity at 40 and 50 min after arabinose induction (Figure 1D and Figure 2A).

### 2.2. CsrB Affects MiaA mRNA Levels and Translation

We transformed the ΔP*_BAD_*-*miaA*-*lacZ* translational fusion strain with pBR-pLac or pBR-*csrB* to execute a complementation assay (Figure 2A). We did observe complementation of the *csrB* mutant with the *csrB* plasmid. CsrB repression of *miaA*-*lacZ* was slightly more pronounced in the *csrB*^−^ background, causing a 7-fold vs. 5-fold inhibition (Figure 2A). We then measured the steady-state levels of MiaA mRNA upon over-expression of CsrB and McaS. We decided to measure the effect of McaS on MiaA mRNA levels since it also acts to sequester CsrA [66] (Figure 2B). Consistent with decreased activity of the P*_BAD_*-*miaA*-*lacZ* translational fusion, we observed a decrease in the *miaA* transcript level upon over-expression of CsrB (Figure 2B,C), with CsrB proving to be a much more effective regulator of the *miaA* RNA level. However, over-expression of McaS did not affect *miaA* mRNA levels (Figure 2B,C).

### 2.3. MiaA Is Regulated at the Level of mRNA Stability by PNPase and RNase E

**Figure 3:**
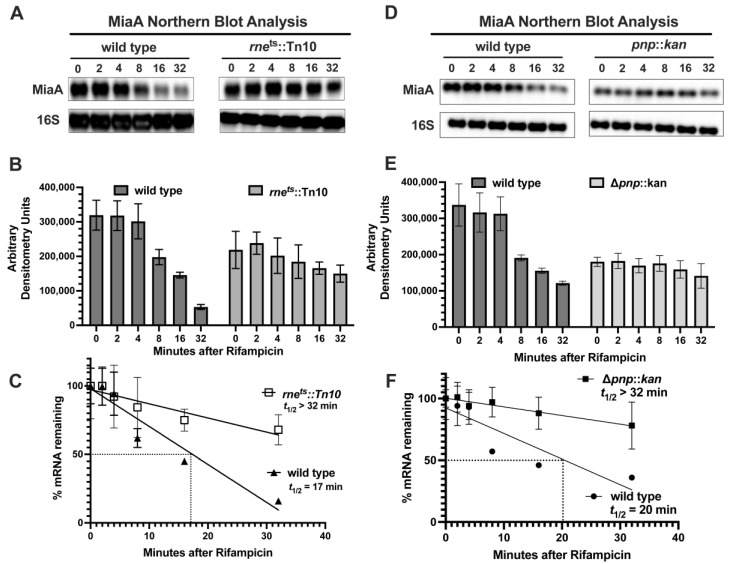
Effects of RNase E and PNPase on *miaA* mRNA stability. (**A**) Northern blot analysis of *miaA* mRNA stability. A temperature-sensitive mutant RNase E (*rne-3071 zce-726*::Tn*10*) allele was transduced from KMT621 into MG1655 (KMT665) by bacteriophage P1 transduction and selected for tetracycline (Tet^R^) resistance KMT801. Wild-type and *rne^ts^* strains were grown in rich media (LB) at 30 °C to an OD_600_ of 0.3. Sample aliquots of 600 μL were collected for total RNA isolation and Northern blot analysis at zero minutes, before transferring cultures to 43 °C. Rifampicin was added, and the samples were collected at 2, 4, 8, 16, and 32 min for total RNA isolation and analysis by agarose Northern blot. Experiments were repeated at least three times and the blots shown are representative blots of the triplicate experiments. (**B**) Quantitative densitometry of Northern blot in Section (**A**). Statistical analysis includes mean and standard error of the mean (mean ± s.e.m.). (**C**) Half-life calculations of Northern blot executed in Section (**A**). Quantitative Northern blot data from Section (**B**) were subjected to linear regression analysis using GraphPad Prism 9. (**D**) Δ*pnp*::*kan* mutation was transduced from KMT624 into MG1655 (KMT665) by bacteriophage P1 transduction and selected for kanamycin (kan^R^) resistance (KMT800). Wild-type and *pnpA*^−^ strains (KMT665 and KMT800) were grown in rich media (LB) to an OD_600_ of 0.3. Then, 600 μL aliquots of the sample were collected for total RNA isolation and Northern blot analysis at zero minutes. Rifampicin was added, and samples were collected at 2, 4, 8, and 16 min for total RNA isolation and analysis by agarose Northern blot. (**E**) Quantitative densitometry of Northern blot in Section (**A**). Statistical analysis includes mean and standard error of the mean (mean ± s.e.m.) (**F**) Half-life calculations of Northern blot executed in Section (**D**). Quantitative Northern blot data from Section (**B**) were subjected to linear regression analysis using GraphPad Prism 9.

Since very little is known regarding the regulation of MiaA mRNA stability, we decided to test the role of RNases in *miaA* mRNA turnover. Previous studies demonstrated an increase in mRNA levels of the *miaA* operon under non-permissive conditions in a temperature-sensitive RNase E mutant [31]. We tested the roles of RNase E and PNPase, both of which are components of the RNA Degradosome, in *miaA* mRNA turnover. Specifically, we compared *miaA* mRNA recycling in the cell, in WT isogenic wild-type, PNPase mutant (*pnpA*^−^), and temperature-sensitive RNase E mutants (*rne^ts^*) (Figure 3). The wild-type and *rne^ts^* strains were grown at 32 °C to mid-log phase and then shifted to 43.5 °C, the non-permissive condition for the RNase E *ts* mutant. Then, we immediately added rifampicin to the cultures to halt transcription. We then isolated total RNA at times of 0, 2, 4, 8, 16, and 32 min after rifampicin treatment and measured *miaA* mRNA levels by Northern blot (Figure 3A,B). Upon semi-quantitative densitometric analysis of the Northern blot analysis and statistical analysis of the *miaA* mRNA in this experiment (Figure 3B,C), we determined that the *t*_1/2_ of the *miaA* mRNA increased in the *rne*^ts^ (>32 min) vs. the wild-type control (17 min) (Figure 3C). We grew wild-type and Δ*pnpA*::*kan* mutants in rich media at 37 °C to mid-log, added rifampicin, and isolated total RNA at 0, 2, 4, 8, 16, and 32 min post-rifampicin treatment (Figure 3D,E). The *t*_1/2_ of the *miaA* mRNA was >32 min and 20 min in the Δ*pnpA*::*kan* and wild-type genetic backgrounds, respectively (Figure 3E,F). The longer half-life of the *miaA* transcript in the *rne*^ts^ allele and Δ*pnpA*::*kan* mutant, in comparison to the wild-type control, suggests that RNase E and PNPase are involved in the post-transcriptional regulation of *miaA* at the level of mRNA stability.

### 2.4. CsrA Is Necessary for the Full Expression of MiaA

**Figure 4:**
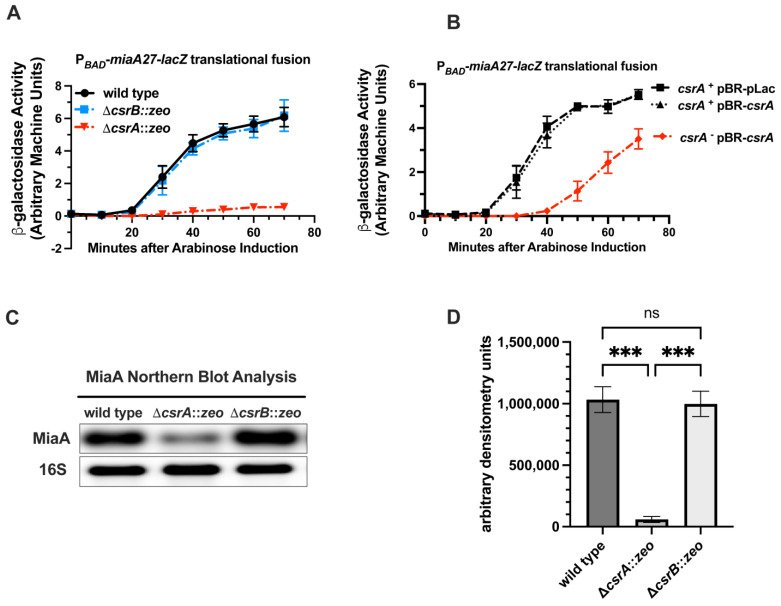
The expression of *miaA* in the absence of *csrA*. (**A**) Wild-type, Δ*csrA*::*zeo,* and Δ*csrB*::*zeo* versions of P*_BAD_*-*miaA27*_(P2HS)_-*lacZ* translational fusions (JIA4000, JIA4044, and JIA4042, respectively) were grown in LB supplemented with glucose to an OD_600_ of 0.5, and then shifted to LB supplemented with arabinose; aliquots were obtained for β-galactosidase assay every 10 min for 70 min. (**B**) CsrA complementation assay using P*_BAD_*-*miaA27*_(P2HS)_-*lacZ* translational fusion activity. Wild-type (*csrA*^+^) and Δ*csrA*::*zeo* (*csrA*^−^) strains of P*_BAD_*-*miaA27*_(P2HS)_-*lacZ* fusions carrying pBR-pLac or pBR-*csrA* were grown in rich media (LB) to an OD_600_ of 0.5 and shifted to LB supplemented with arabinose; aliquots were obtained for β-galactosidase assay every 10 min for 70 min. (**C**) Northern blot analysis of *miaA* mRNA from total RNA isolated from exponentially growing wild-type, Δ*csrA*::*zeo,* and Δ*csrB*::*zeo* cells. Experiments were repeated at least three times and blots shown are representative blots of the triplicate experiments. (**D**) Quantitative densitometry of Northern blot analysis in (**C**). Densitometry signals were acquired using Fluorochem R Fluorescent/Chemiluminescent imager and statistical analysis of densitometry was executed using One-Way ANOVA with Tukey’s Multiple Comparisons test on GraphPad Prism 9 (*** *p*-value = 0.001, ns = not significant).

The RNA-binding protein CsrA is part of a global regulatory system that controls bacterial gene expression at the post-transcriptional level [40,41,62,67,68,69,70,71]. CsrA regulates translation of target proteins by binding to target sequences in the 5′ UTR of the target genes. The availability of CsrA is regulated by sequestration of CsrB and CsrC sRNAs. CsrB and CsrC have multiple CsrA binding sites, each binding to approximately 18 CsrA subunits and inhibiting the activity of CsrA. Since CsrB represses the expression of *miaA*, and CsrB acts to sequester and inhibit CsrA activity, we hypothesized that the regulatory effect of CsrB on *miaA* may be through CsrA. To test our hypothesis, we measured the activity of the P*_BAD_*-*miaA27*-*lacZ* translational fusion in a *csrA*^−^ genetic background with over-expression of CsrA (Figure 4). In the *csrA*^−^ background, the β-galactosidase activity of the P*_BAD_*-*miaA27*-*lacZ* strain was decreased by approximately 6–10-fold at 70 min after arabinose induction, and was essentially non-detectable, in comparison to the *csrA*^+^ and *csrB*^−^ genetic backgrounds (Figure 4A). The β-galactosidase activity of the *csrA*^−^ P*_BAD_*-*miaA27*-*lacZ* strain was partially rescued by over-expression of plasmid-based *csrA* (Figure 4B). Given the decrease in the activity of the P*_BAD_*-*miaA27*-*lacZ* fusion, in the *csrA* mutant, we decided to measure *miaA* mRNA levels in the absence of *csrA*. We measured *miaA* mRNA levels in the wild-type, *csrA*^−^, and *csrB*^−^ genetic backgrounds. The *miaA* mRNA levels were decreased, in a statistically significant manner, by approximately 20-fold in the absence of *csrA* while remaining virtually unchanged in the absence of *csrB* (Figure 4C,D). This result is consistent with *miaA* mRNA down-regulation upon over-expression of CsrB (Figure 1C) and confirms that the CsrA-CsrB system regulates post-transcriptional expression of *miaA*.

## 3. Discussion

### 3.1. RNA Modifications and the Bacterial Epitranscriptome

RNA modifications have long been recognized as essential for maintaining translational fidelity and the structural stability of tRNAs and rRNAs. In bacterial systems, parttRNA modifications in particular have been extensively studied for their role in ensuring accurate and efficient protein synthesis [72,73,74,75]. While early work focused on a few bacterial model organisms, recent studies have broadened this view to include diverse species, providing insights into bacterial pathogenesis and the regulation of virulence [76,77,78,79,80]. Additionally, the potential for targeting tRNA modification pathways as antimicrobial strategies is now under active investigation [81,82]. More recent findings have further expanded the scope of the bacterial epitranscriptome to include modifications in mRNAs, suggesting regulatory functions beyond the canonical roles in tRNA and rRNA [83]. Studies in eukaryotic systems have also highlighted an expanded list of RNA species that contain RNA modifications. These include extensive mRNA modifications within the coding and noncoding regions [84]. Beyond mRNAs, RNA modifications have been found within eukaryotic regulatory RNA species such as miRNA, CircRNAs, and lncRNAs [85,86,87]. They are thought to play roles in post-transcriptional regulatory circuits by modulating miRNA interactions with the 3′ UTRs of mRNAs, modulating mRNA stability. Several different RNA modifications play regulatory roles in cellular physiology [88,89,90,91,92,93]. Work by our group and others has demonstrated that RNA modifications may play regulatory roles in gene expression by promoting the expression of stress response genes, in a manner dependent upon codon bias in bacteria and bacteriophages [25,26,27,94,95,96]. Subsequent reports from several groups have also demonstrated codon-biased gene regulation in bacterial pathogens [25,26,27,95,96]. In all these studies, the role of the modification was established by mutating the gene(s) encoding the modification enzymes. To add to these discoveries, understanding the conditions whereby RNA modifications are synthesized will assist us in understanding their impact on the physiology of the cell.

### 3.2. New Regulators of MiaA Expression

We previously demonstrated that *miaA* is necessary for the expression of RpoS, IraP, and Hfq [25,26,27]. The *miaA* requirement for optimal expression of RpoS and IraP is related to UUX-leucine decoding, suggesting that MiaA may promote the expression of stress response genes during leucine starvation [25,26,27]. Over-expression of leucine tRNAs was able to suppress the decreased expression of *rpoS* in *miaA* mutants. Interestingly, a previous study demonstrated that *miaA* mutants are synthetically lethal with *leuX* (leucine tRNA) mutants during heat shock [97]. Also, *miaA* mutants affect leucine operon gene expression in Salmonella [4]. Taken together, this suggests that MiaA-catalyzed i^6^A37 modification is particularly important during heat shock whereby UUX-leucine decoding provides an adaptive advantage. For these reasons, characterizing the post-transcriptional regulation of the *miaA* transcript from the σ^32^-dependent heat shock promoter is of critical importance. Prior to this work, little was known about post-transcriptional regulation of *miaA*. Here, we started to fill that knowledge gap by identifying several potential candidate sRNA regulators of MiaA expression at the post-transcriptional level. Our results demonstrate a strong effect of CsrA, likely as a direct post-transcriptional regulator of the *miaA*_P2_ promoter transcript.

### 3.3. MiaA Is an Additional Potential Stimulatory Target of the CsrA-CsrB System and Interactions with Other Post-Transriptional Regulators of the MiaA Operon

**Figure 5:**
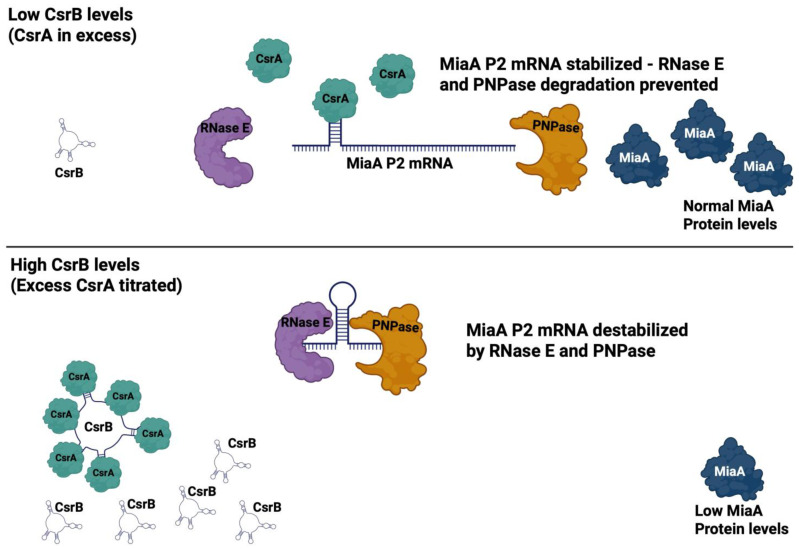
Model for CsrA-CsrB regulation of *miaA* expression. Graphical model of CsrA/CsrB regulation of MiaA was created using Biorender.com. MiaA transcript turnover is mediated through both PNPase and RNase E. CsrA promotes the accumulation of the *miaA* P2 (heat shock) (HS) mRNA in the absence of CsrB. In the presence of CsrB, CsrA sequestration leads to decreased levels of the *miaA* P2 (heat shock) mRNA.

CsrA can directly bind to mRNA transcripts to regulate gene expression at the post-transcriptional level, in the absence of sequestration by CsrB or CsrC [40]. There are approximately 12 mRNA transcripts that are post-transcriptionally regulated by CsrA. Ten of these regulatory targets are repressed and two of these regulatory targets are stimulated by CsrA, including *ymdA* [98] (Table 1). CsrA stimulates the translation of *ymdA* through interaction with its 5′ UTR to reverse the formation of secondary structures that occlude its ribosome binding site and subsequent translational initiation. Our work identifies *miaA* as a stimulatory target of CsrA. It is possible that the 5′ UTR of the *miaA*_P2_ transcript has secondary structures that occlude the ribosome binding site and that CsrA acts in a similar manner on this transcript to stimulate translation of this transcript. Given the fact that RNase E and PNPase mutants result in the stabilization of the *miaA* transcript, CsrA may also interact with Degradosome enzymes or the 5′ untranslated region (UTR) of the MiaA P2 transcript to promote its stabilization. We have illustrated a simple model to describe CsrA-CsrB regulation of *miaA* (Figure 5). Upon sequestration of CsrA by CsrB, the *miaA* transcript from the P2 (heat shock promoter) is less stable and translation is also inhibited. This is reversed in the absence of CsrB, and when CsrA levels are higher or it is more active, resulting in transcript stabilization and an increase in translation (Figure 5). The stabilization of the *miaA* mRNA could be through antagonistic interactions with Degradosome proteins RNase E and PNPase. This model is consistent with the previous report from Vakulskas et al. (2016), whereby CsrA was shown to stabilize CsrB through antagonistic interactions with RNase E [99].

The *miaA* gene is encoded directly upstream of the *hfq* gene within the *nnr*-*tsaE*-*amiB*-*mutL*-*miaA*-*hfq*-*hflX*-*hflK*-*hflC* superoperon in the *E. coli* genome [30,31,100,101]. This tandem genetic localization is widely conserved across the prokaryotic domain. While mutations in *hfq* exhibit extensively pleiotropic effects, *miaA* mutant phenotypes appear to be distinct, apart from their effects on *rpoS* expression, whereby the *miaA* mutant demonstrates some polarity likely due to effects on Hfq-dependent sRNAs that stimulate *rpoS* translation [25,102,103,104,105,106,107]. There is complex transcriptional organization of this superoperon with approximately 10 promoters exhibiting alternating dependence upon σ^32^ an σ^70^, in the *amiB*-*hfq* region [30,31,100,101]. All these promoters are upstream of the *hfq* gene. However, there are three promoters between the *miaA* gene and the start codon of *hfq*, with the function of drive expression of *hfq*-*hflXKC*: defined as *hfq*_P1(hs)_, *hfq*_P3_, and *hfq*_P3_. Baker et al. (2007) demonstrated that CsrA binds to the ribosome binding site of the *hfq* mRNA and inhibits *hfq* translation [108]. In our screen, we utilized a *miaA*-*lacZ* fusion with the *miaA* P2 transcript. If CsrA interacts with the 5′ UTR of the *miaA* P2 transcript, it may provide CsrA with multiple options for binding to the larger transcripts expressed from this operon that contains both *miaA* and *hfq*. Multiple CsrA binding sites may or may not be accessible on the larger transcripts. This is the subject of ongoing studies in our laboratory.

## 4. Materials and Methods

### 4.1. Strains and Plasmids

All strains are derivatives of *E. coli* K12 MG1655 and are listed in Table 2. All plasmids used in this study are also listed in Table 2.

### 4.2. Media and Growth Conditions

*E. coli* strains were grown in Luria–Bertani (LB) Lennox liquid media (KD Medical, Columbia, MD, USA) in a WS27 shaking water bath (ShelLab, Cornelius, OR, USA) for the experiments in this study at 37 °C, unless otherwise described below. Transformation of plasmid DNA into strains was sometimes facilitated using Transformation Storage Solution (TSS) media (LB, 10% (*w*/*v*) Polyethylene Glycol-8000, 5% DMSO, 50 mM MgCl_2_) as described below. Recombinants from Lambda (λ)-Red-based mutagenesis were selected for growth on LB agar plates supplemented with zeomycin to a final concentration of 25–50 μg/mL (LB-Zeo). Transductants, of *pnpA*::*kan* or *rne-3071 zce-726*::Tn*10* (*tet*) mutants, were grown on LB agar plates supplemented with either tetracycline or kanamycin to a final concentration of 25 μg/mL (LB-Tet or LB-Kan). The *rne-3071 zce-726*::Tn*10* mutant is temperature-sensitive and requires incubation at 30 °C for optimal growth. To determine the effect of RNase E on *miaA* mRNA levels, *rne-3071 zce-726*::Tn*10* cultures were grown at 30 °C and then shifted to 43.5 °C to induce an RNase E^−^ phenotype, as previously described [36]. We then isolated total RNA for the analysis of *miaA* mRNA levels or turnover using Northern blot analysis. Strains carrying plasmids were grown in LB media supplemented with ampicillin to a final concentration of 100 μg/mL (LB-Amp) or on LB-Amp agar plates. To stimulate the expression of arabinose-inducible fusion strains, cultures were first grown in LB or LB-Amp, supplemented with glucose to a final concentration of 0.2%, harvested by centrifugation, washed once with LB media, and resuspended in LB supplemented with arabinose to a final concentration of 0.2%. Small RNA plasmid library transformants were assayed on MacConkey-Lactose (Mac-Lac) agar plates supplemented with ampicillin to a final concentration of 100 µg/mL (Mac-Lac-Amp) to screen for changes in the Lactose phenotype of individual colonies following overnight growth at 37 °C.

### 4.3. General Molecular Biology Techniques

Plasmid DNA was isolated using The Column-Pure^TM^ Plasmid Mini-Prep Kit (Lamda Biotech, St. Louis, MO, USA), according to manufacturer instructions. Genomic DNA used for PCR reactions was isolated using The Column-Pure^TM^ Bacterial Genomic DNA Kit (Lamda Biotech), according to the manufacturer’s instructions. PCR reactions were performed to amplify allelic exchange substrates for mutagenesis. PCR amplification was performed using the Taq Plus 2X PCR MasterMix (Lamda Biotech) with standard cycle conditions according to the manufacturer’s instructions. The annealing temperature for each PCR reaction was optimized to the T_m_ of the primers used (Table 3). All PCR reactions were purified using The Column-Pure^TM^ Clean-Up Kit (Lamda Biotech), according to the manufacturer’s instructions (Lamda Biotech).

### 4.4. Genetic Engineering and Strain Construction

Chromosomal mutagenesis was executed via bacteriophage λ-based recombineering and bacteriophage P1 transduction as previously described and outlined below [109,110]. Plasmids were moved using the Transformation Storage Solution (TSS) media protocol or heat shock transformation of chemically competent cells as described below [111].

#### 4.4.1. Insertional Inactivation Mutagenesis Using Recombineering

Deletion and insertion mutations of *csrA*, *csrB*, and *miaA* were constructed using recombineering as previously described [110,112]. Briefly, a zeomycin resistance cassette with 50 bp of flanking homology to *csrA*, *csrB*, or *miaA* was synthesized by PCR to create an allelic exchange substrate for recombineering-based mutagenesis. Then, the DJ480 mini-λ::*tet* strain was induced using heat shock and made electrocompetent with ice-cold water washes [110,112]. The allelic exchange substrates were then electroporated into the prepared cells, allowed to recover, and plated on LB-Zeo selectable media. Recombinants were confirmed to have the mutant using PCR with a primer upstream or downstream of the target gene along with a primer that recognizes the insertion marker. Finally, this was verified using Sanger Sequencing.

#### 4.4.2. P1 Transduction to Move Mutants Between Strains

The newly constructed Δ*csrA*::*zeo*, Δ*csrB*::*zeo*, and Δ*miaA*::*zeo* mutations, as well as the previously constructed *pnpA*::*kan* and *rne-3071 zce-726*::*Tn10* (*tet*) mutants, were moved from the mini-λ::*tet*-containing strain into clean genetic backgrounds (MG1655 or P*_BAD_*-*miaA27*_P2_-*lacZ* translational fusion strain) via generalized transduction using bacteriophage P1 as previously described [113].

#### 4.4.3. Heat Shock Transformation of Chemically Competent Cells for Cloning

All plasmids, including those from the sRNA library, were transformed into *E. coli* strains using Transformation Storage Solution (TSS) media and its associated transformation method as previously described [111]. Briefly, the *E. coli* K-12 MG1655 recipient strain was grown in nutrient-rich Lennox Broth (LB) to OD_600_ 0.5. Upon reaching OD_600_ 0.5, the cells were harvested by centrifugation. The supernatant was then decanted, and the pellet was re-suspended in 1/10th volume of ice-cold TSS media. An amount of 2 μL (100 ng) of plasmid DNA (pBR-pLac, pBR-pLac-*csrB*, pBR-pLac-*sdsR*, pBR-pLac-*spf*, pBR-pLac-*arcZ*, pBR-pLac-*gcvB*, pBR-pLac-*mcaS*, and pBR-pLac-*csrA*) was added to 100 μL aliquots of cell suspension and incubated on ice for 30 min. Following the 30 min incubation on ice, 900 μL of LB was added to the cell–plasmid mixture, and the mixture was left to recover at 37 °C for 1 h on a shaking heat block. Upon the completion of the recovery period, 200 μL of recovered cells was plated on LB-Amp plates and left to grow overnight at 37 °C in a microbiological incubator. Transformants were purified once by streaking on LB agar plates supplemented with ampicillin.

### 4.5. RNA Isolation

Total RNA was isolated using the Hot Phenol Method as previously described [114,115]. Briefly, overnight cultures were subcultured in 30 of LB at a 1:1000 dilution ratio and allowed to grow in the shaking water bath to an Optical Density of 600 (OD_600_) of 0.5. A 600 μL aliquot of cells was isolated from exponentially growing *E. coli* cultures and resuspended in a 1× lysis buffer/Hot Acid Phenol solution in a 1.5 mL microcentrifuge tube on a thermomixer (Eppendorf, Hamburg, Germany) set to 65 °C. The cells were incubated with intermittent shaking for 5 min. The tubes were subjected to centrifugation at 15,000 rpm for 10 min. The aqueous phase was extracted and purified two additional times with acid-phenol followed by ethanol precipitation in a −80 °C freezer overnight. The RNA was pelleted and washed with 70% ethanol, air-dried, and resuspended with 50 μL of DEPC water. RNA concentrations were successively measured using The NanoDrop^TM^ One^C^ Microvolume UV-Vis Spectrophotometer (Fisher Scientific, Waltham, MA, USA).

### 4.6. Agarose Northern Blot

The agarose-based Northern blot was executed as previously described [36,116]. A 1× MOPS (Quality Biological INC, Gaithersburg, MD, USA) 1% Agarose gel was used for the resolution of total RNA. After pre-running the gel at 100 V for 40 min, a constant amount of total RNA (ranging from 2 to 5 μg) was mixed with 2× volume of loading buffer (500 μL Formamide, 100 μL 10× MOPS, 100 μL (80% glycerol 0.2% bromophenol blue), 120 μL Formaldehyde, and 2 μL (10 mg/mL EtBr)). The samples were then heated at 65 °C for 15 min and loaded onto the gel for fractionation by gel electrophoresis for 40 min at 100 volts. The gel was then soaked in 0.05 M NaOH solution for 20 min and 20× SSC solution for 1 h. The RNA was then transferred from the agarose gel to a nylon membrane using the capillary method as previously described [117]. The RNA was then UV-crosslinked to the nylon membrane using the HL-2000 Hybrilinker (UVP/Analytikjena, Upland, CA, USA). After UV-crosslinking of the nylon membrane, it was then pre-hybridized with 5 mL of ultrahyb oligo buffer (Ambion, Austin, TX, USA) for 2 h, and then subjected to hybridization with a biotinylated DNA probe targeting the transcript of interest overnight at 42 °C in the HL-2000 Hybrilinker (UVP). The membranes were then processed using stringency washes and developed using the Chemiluminescent Nucleic Acid Detection Module Kit (ThermoFisher Scientific, Waltham, MA, USA) according to the manufacturer’s recommendations.

### 4.7. β-Galactosidase Assays (Kinetic Microtiter Assays)

We measured β-galactosidase activity using the Kinetic Microtiter Plate Assay method [118]. Briefly, overnight cultures were grown in 5 of LB Lennox liquid media at 37 °C within a Cel-Gro Tissue culture rotator (Thermo Scientific) placed within a microbiological incubator (ShelLab). Overnight cultures containing sRNA plasmids were grown in LB-Amp. The following day, the overnight cultures were diluted 1:1000 in 30 mL of fresh LB or LB-Amp liquid media and placed in a 125 mL beveled Erlenmeyer flask to sub-culture the cells. IPTG was added to cultures containing sRNA plasmids to a final concentration of 1 mM. Cultures were then grown in a WS27 shaking water bath (ShelLab) at 37 °C to an OD_600_ of 0.5. Cells were then harvested by centrifugation and then washed and resuspended with Lennox Broth (LB). The LB–cell suspensions were then transferred to a new flask and supplemented with ampicillin (100 μg/mL), IPTG (1 mM), and arabinose (0.02%). The cultures were then incubated in the 37 °C shaking water bath for an additional 70 min. Then, 100 μL aliquots of the cultures were collected once, or at 10 min intervals, and transferred to a 96-well polystyrene microtiter plate containing 50 μL of permeabilization solution (100 mM of Tris, pH 7.8, 32 mM of NaPO_4_, 8 mM of DTT (Dithiothreitol), 8 mM CDTA (Cyclohexanediaminetetraacetic acid), 4% Triton X-100, and 50 μL of Polymixin B). To serve as an experimental control, 100 μL of LB was pipetted into 1 of the wells of the 96-well polystyrene microtiter plate. The 96-well polystyrene microtiter plate containing the samples was incubated at room temperature for 15 min to allow for cell lysis. Subsequently, 50 μL of O-nitrophenyl-β-D-galactoside (ONPG) solution (4 mg/mL ONPG, 2 mM of Sodium Citrate, and 70 μL of β-Mercaptoethanol) was added to each well containing cell lysates. The 96-well polystyrene microtiter plate was immediately read using a Filter Max F5 Multi-Mode Micro Plate Reader (Molecular Devices, San Jose, CA, USA).

Arbitrary machine units were assigned to each sample, calculated as OD_420_/OD_600_. These units are approximately 25-fold lower than the Miller units classically used for β-galactosidase assays.

### 4.8. Statistical Analysis

All statistical analysis was executed using Prism 9 software (Graphpad, La Jolla, CA, USA).

## Figures and Tables

**Table 1:** CsrA regulation.

Repressed	Activated
*glgC*	*moaABC operon*
*glgS*	*ymdAB-clsC*
*sdiA*	
*dgcZ*	
*dgcT*	
*pgaA*	
*pdeI*	
*nhaR*	
*rpoE*	
*iraD*	

**Table 2:** Strains and plasmids.

Strain Number	Genotype	Construction, Source, or Comment
DY330	W3110 λ*lacU169 gal490 pgl* l8 [λ *c*I*857* λ(*cro-bioA*)]	62
JIA4000	MG1655 Δ*araBAD*, *araC*^+^, *mal*::*lacI*^q^ Φ80*^−^ lacI*::*P_BAD_-miaA27P2-lacZ* translational fusion	KMT590 × P*_BAD_*-*miaA27*_(P2HS)_-*lacZ* gBlock
JIA4001	MG1655 Δ*araBAD*, *araC*^+^, *mal*::*lacI*^q^ Φ80*^−^ lacI*::*P_BAD_-miaA27P2-lacZ* translational fusion pBR-pLac	JIA4000 + pBR-pLac
JIA4010	MG1655 Δ*araBAD*, *araC*^+^, *mal*::*lacI*^q^ Φ80*^−^ lacI*::*P_BAD_-miaA27P2-lacZ* translational fusion pBR-*sdsR*	JIA4000 + pBR-*sdsR*
JIA4015	MG1655 Δ*araBAD*, *araC*^+^, *mal*::*lacI*^q^ Φ80*^−^ lacI*::*P_BAD_-miaA27P2-lacZ* translational fusion pBR-*gcvB*	JIA4000 + pBR-*gcvB*
JIA4018	MG1655 Δ*araBAD*, *araC*^+^, *mal*::*lacI*^q^ Φ80*^−^ lacI*::*P_BAD_-miaA27P2-lacZ* translational fusion pBR-*arcZ*	JIA4000 + pBR-*arcZ*
JIA4024	MG1655 Δ*araBAD*, *araC*^+^, *mal*::*lacI*^q^ Φ80*^−^ lacI*::*P_BAD_-miaA27P2-lacZ* translational fusion pBR-*spf*	JIA4000 + pBR-*spf*
JIA4029	MG1655 Δ*araBAD*, *araC*^+^, *mal*::*lacI*^q^ Φ80*^−^ lacI*::*P_BAD_-miaA27P2-lacZ* translational fusion pBR-*csrB*	JIA4000 + pBR-*csrB*
JIA4040	MG1655 Δ*araBAD*, *araC*^+^, *mal*::*lacI*^q^ Φ80*^−^ lacI*::*P_BAD_-miaA27P2-lacZ* translational fusion ∆*arcZ*::*zeo*	JIA4000 × P1 (KMT657)
JIA4041	MG1655 Δ*araBAD*, *araC*^+^, *mal*::*lacI*^q^ Φ80*^−^ lacI*::*P_BAD_-miaA27P2-lacZ* translational fusion ∆*gcvB*::*kan*	JIA4000 × P1 (KMT660)
JIA4042	MG1655 Δ*araBAD*, *araC*^+^, *mal*::*lacI*^q^ Φ80*^−^ lacI*::*P_BAD_-miaA27P2-lacZ* translational fusion ∆*csrB*::*zeo*	JIA4000 × P1 (DJ480 ∆*csrB*::*zeo*)
JIA4043	MG1655 Δ*araBAD*, *araC*^+^, *mal*::*lacI*^q^ Φ80*^−^ lacI*::*P_BAD_-miaA27P2-lacZ* translational fusion ∆*spf*::*cat*	JIA4000 × P1 (KMT658)
JIA4044	MG1655 Δ*araBAD*, *araC*^+^, *mal*::*lacI*^q^ Φ80*^−^ lacI*::*P_BAD_-miaA27P2-lacZ* translational fusion ∆*csrA*::*zeo*	JIA4000 × P1 (DJ480 ∆*csrA*::*zeo*)
JIA4045	MG1655 Δ*araBAD*, *araC*^+^, *mal*::*lacI*^q^ Φ80*^−^ lacI*::*P_BAD_-miaA27P2-lacZ* translational fusion ∆*sdsR*::*kan*	JIA4000 × P1 (KMT662)
KMT195	DJ480 mini- λ::*tet*	NM300 obtained from Susan Gottesman Lab, NCI-NIH
KMT414	MG1655 Δ*lacZ*YA*_frtfrt_ lacI^q^* ∆a*ra714* ∆P*_araE_*::*_frtfrt_*P_CP18_*araE*	Thompson Lab collection
KMT621	*C600 rne3071::Tn10*-*Tet^R^*	C600 × P1 (*rne3071*::Tn10-Tet), temperature-sensitive
KMT624	MG1655 Δ*pnp*::*kan*	NRD465, cold-sensitive, obtained from Gottesman Lab, NCI-NIH
KMT657	MG1655 Δ*arcZ*::*zeo*	NM665 (NM1100 ∆*arcZ*::*zeo*) gift from Nadim Majdalani-Gottesman Lab, NCI-NIH
KMT658	W3110 λ *lacU169 gal490 pgl* l8 [λ *c*I*857* λ (*cro-bioA*)] ∆*spf*::*cat*	NM18 (DY330 ∆*spf*::*cat*) gift from Nadim Majdalani-Gottesman Lab, NCI-NIH
KMT660	MG1655 *mal*::*IacI*^q^ Δ*araBAD leu*^+^ *araC*^+^ ∆P*_araE_::_frtfrt_P_CP18araE_*	KM357 (PM101 ∆*gcvB*::*kan*) obtained from Gottesman Lab, NCI-NIH
KMT662	MG1655 Δ*sdsR*::*kan*	ASP7023 (∆*sdsR*::*kan*) obtained from Gottesman Lab, NCI-NIH
KMT665	MG1655 *lacI*^q^	NM525 obtained from Gottesman Lab, NCI-NIH
KMT719	MG1655 *lacI*^q^ ∆*csrB*::*zeo*	KMT665 × P1 (DJ480 ∆*csrB*::*zeo*)
KMT720	MG1655 *lacI*^q^ ∆*csrA*::*zeo*	KMT665 × P1 (DJ480 ∆*csrA*::*zeo*)
KMT791	MG1655 *lacI*^q^ pBR-*csrA*	KMT665 + pBR-*csrA* (TSS Transformation)
KMT792	MG1655 *lacI*^q^ pBR-*csrB*	KMT665 + pBR-*csrB* (TSS Transformation)
KMT796	MG1655 *lacI*^q^ ∆*csrB*::*zeo* pBR-pLac-*csrB*	KMT719 + pBR-*csrB* (TSS Transformation)
KMT798	MG1655 *lacI*^q^ ∆*csrA*::*zeo* pBR-pLac-*csrA*	KMT720 + pBR-*csrA* (TSS Transformation)
KMT799	MG1655 *lacI*^q^ pBR-pLac-*mcaS*	KMT665 + pBR-*mcaS* (TSS Transformation)
KMT800	MG1655 *lacI*^q^ ∆*pnpA*::*kan*	KMT665 × P1 (KMT624-∆*pnp*::*kan*)
KMT801	MG1655 *lacI*^q^ *rne3071*::*Tn10* (Tet^R^)	KMT665 × P1 (*C600 rne3071::Tn10-Tet^R^*)
KMT590	MG1655 *lacI*::P*_BAD_*::*cat*-*sacB*::*lacZ*, Δ*araBAD*, *araC*^+^, *mal*::*lacI*^q^, mini- λ::*tet* Φ80^−^	PM1805 obtained from Nadim Majdalani-Gottesman Lab, NCI-NIH

**Table 3:** Oligonucleotide primers and probes.

Oligo Primer	Sequence (5′ to 3′ Orientation)	Purpose Description
KT1205	5′-tcgacgtcCTTTCAAGGAGCAAAGAatgCTGAT-3′	*csrA* into pBR-pLac AatII
KT1206	5′-tcagaattcttaGTAACTGGACTGCTGGG-3′	*csrA* into pBR-pLac EcoRI
KT1207	5′-CAGAGAGACCCGACTCTTTTAATCTTTCAAGGAGCAAAGA-3′	Δ*csrA*::*zeo* forward screening
KT1208	5′-TGAGGGTGCGTCTCACCGATAAAGATGAGACGCGGAAAGA-3′	Δ*csrA*::*zeo* reverse screening
KT1209	5′-CAGAGAGACCCGACTCTTTTAATCTTTCAAGGAGCAAAGACACGTGTTGACAATTAATCA-3′	Δ*csrA*::*zeo* forward mutagenesis
KT1210	5′-TGAGGGTGCGTCTCACCGATAAAGATGAGACGCGGAAAGATCAGTCCTGCTCCTCGGCCA-3′	Δ*csrA*::*zeo* reverse mutagenesis
KT1211	5′-GCGCCTTGTAAGACTTCGCGAAAAAGACGATTCTATCTTC-3′	Δ*csrB*::*zeo* forward screening
KT1212	5′-AGCAACCTCAATAAGAAAAACTGCCGCGAAGGATAGCAGG-3′	Δ*csrB*::*zeo* reverse screening
KT1213	5′-GCGCCTTGTAAGACTTCGCGAAAAAGACGATTCTATCTTCCACGTGTTGACAATTAATCA-3′	Δ*csrB*::*zeo* forward mutagenesis
KT1214	5′-AGCAACCTCAATAAGAAAAACTGCCGCGAAGGATAGCAGGTCAGTCCTGCTCCTCGGCCA-3′	Δ*csrB*::*zeo* reverse mutagenesis
MiaA probe	5′-Biosg/CGCGGCGAGTAACTCTTCAGCGTTCGGCTTCGCCG-3′	Biotinylated oligo antisense to miaA
16S probe	5′-Biosg/CACAACACGAGCTGACGACAGCCATGCAGCACCTG-3′	Biotinylated oligo antisense to 16S rrnA

## Data Availability

Data is contained within the article.

## References

[1] Caillet J., Droogmans L. (1988). Molecular cloning of the *Escherichia coli miaA* gene involved in the formation of delta 2-isopentyl adenosine in tRNA. J. Bacteriol.

[2] Connolly D.M., Winkler M.E. (1989). Genetic and physiological relationships among the *miaA* gene, 2-methylthio-N6-(delta 2-isopentenyl)-adenosine tRNA modification, and spontaneous mutagenesis in *Escherichia coli* K-12. J. Bacteriol..

[3] Connolly D.M., Winkler M.E. (1991). Structure of *Escherichia coli* K-12 *miaA* and characterization of the mutator phenotype caused by *miaA* insertion mutations. J. Bacteriol.

[4] Blum P.H. (1988). Reduced *leu* operon expression in a *miaA* mutant of *Salmonella typhimurium*. J. Bacteriol.

[5] Diaz I., Ehrenberg M., Kurland C.G. (1986). How do combinations of *rpsL*- and *miaA*- generate streptomycin dependence?. Mol. Gen. Genet. MGG.

[6] Pierrel F., Bjork G.R., Fontecave M., Atta M. (2002). Enzymatic modification of tRNAs: MiaB is an iron-sulfur protein. J. Biol. Chem..

[7] Esberg B., Leung H.C., Tsui H.C., Bjork G.R., Winkler M.E. (1999). Identification of the *miaB* gene, involved in methylthiolation of isopentenylated A37 derivatives in the tRNA of Salmonella typhimurium and *Escherichia coli*. J. Bacteriol..

[8] Tukenmez H., Xu H., Esberg A., Bystrom A.S. (2015). The role of wobble uridine modifications in +1 translational frameshifting in eukaryotes. Nucleic Acids Res..

[9] Bjork G.R., Durand J.M., Hagervall T.G., Leipuviene R., Lundgren H.K., Nilsson K., Chen P., Qian Q., Urbonavicius J. (1999). Transfer RNA modification: Influence on translational frameshifting and metabolism. FEBS Lett..

[10] Bjork G.R., Wikstrom P.M., Bystrom A.S. (1989). Prevention of translational frameshifting by the modified nucleoside 1-methylguanosine. Science.

[11] Urbonavicius J., Qian Q., Durand J.M., Hagervall T.G., Bjork G.R. (2001). Improvement of reading frame maintenance is a common function for several tRNA modifications. EMBO J..

[12] Urbonavicius J., Stahl G., Durand J.M., Ben Salem S.N., Qian Q., Farabaugh P., Bjork G.R. (2003). Transfer RNA modifications that alter +1 frameshifting in general fail to affect −1 frameshifting. RNA.

[13] Qian Q., Bjork G.R. (1997). Structural requirements for the formation of 1-methylguanosine in vivo in tRNA(Pro)GGG of *Salmonella typhimurium*. J. Mol. Biol..

[14] Qian Q., Bjork G.R. (1997). Structural alterations far from the anticodon of the tRNAProGGG of *Salmonella typhimurium* induce +1 frameshifting at the peptidyl-site. J. Mol. Biol..

[15] Qian Q., Curran J.F., Bjork G.R. (1998). The methyl group of the N6-methyl-N6-threonylcarbamoyladenosine in tRNA of *Escherichia coli* modestly improves the efficiency of the tRNA. J. Bacteriol..

[16] Zhao J., Leung H.E., Winkler M.E. (2001). The *miaA* mutator phenotype of *Escherichia coli* K-12 requires recombination functions. J. Bacteriol..

[17] Schweizer U., Bohleber S., Fradejas-Villar N. (2017). The modified base isopentenyladenosine and its derivatives in tRNA. RNA Biol..

[18] Nishii K., Wright F., Chen Y.Y., Moller M. (2018). Tangled history of a multigene family: The evolution of *ISOPENTENYLTRANSFERASE* genes. PLoS ONE.

[19] Soman S., Ram S. (2022). *MiaA* (Rv2727c) mediated tRNA isopentenylation of *Mycobacterium tuberculosis* H37Rv. Mol. Biol. Res. Commun..

[20] Fleming B.A., Blango M.G., Rousek A.A., Kincannon W.M., Tran A., Lewis A.J., Russell C.W., Zhou Q., Baird L.M., Barber A.E. (2022). A tRNA modifying enzyme as a tunable regulatory nexus for bacterial stress responses and virulence. Nucleic. Acids Res..

[21] Durand J.M., Bjork G.R., Kuwae A., Yoshikawa M., Sasakawa C. (1997). The modified nucleoside 2-methylthio-N6-isopentenyladenosine in tRNA of *Shigella flexneri* is required for expression of virulence genes. J. Bacteriol..

[22] Sun B., Liu H., Jiang Y., Shao L., Yang S., Chen D. (2020). New Mutations Involved in Colistin Resistance in *Acinetobacter baumannii*. mSphere.

[23] Koshla O., Yushchuk O., Ostash I., Dacyuk Y., Myronovskyi M., Jager G., Sussmuth R.D., Luzhetskyy A., Bystrom A., Kirsebom L.A. (2019). Gene *miaA* for post-transcriptional modification of tRNA(XXA) is important for morphological and metabolic differentiation in *Streptomyces*. Mol. Microbiol..

[24] Koshla O., Kravets V., Dacyuk Y., Ostash I., Sussmuth R., Ostash B. (2020). Genetic analysis of *Streptomyces albus* J1074 *mia* mutants suggests complex relationships between post-transcriptional tRNA(XXA) modifications and physiological traits. Folia Microbiol..

[25] Thompson K.M., Gottesman S. (2014). The *MiaA* tRNA modification enzyme is necessary for robust *RpoS* expression in *Escherichia coli*. J. Bacteriol..

[26] Aubee J.I., Olu M., Thompson K.M. (2017). *TrmL* and *TusA* are necessary for *rpoS* and *MiaA* is required for *hfq* expression in *Escherichia coli*. Biomolecules.

[27] Aubee J.I., Olu M., Thompson K.M. (2016). The i6A37 tRNA modification is essential for proper decoding of UUX-Leucine codons during *rpoS* and *iraP* translation. RNA.

[28] Moller T., Franch T., Hojrup P., Keene D.R., Bachinger H.P., Brennan R.G., Valentin-Hansen P. (2002). *Hfq*: A bacterial Sm-like proteins that mediates RNA-RNA interaction. Mol. Cell..

[29] Kajitani M., Kato A., Wada A., Inokuchi H., Ishihama A. (1994). Regulation of the *Escherichia coli hfq* gene encoding the host factor for phage Q beta. J. Bacteriol..

[30] Tsui H.C., Feng G., Winkler M.E. (1996). Transcription of the *mutL* repair, *miaA* tRNA modification, *hfq* pleiotropic regulator, and *hflA* region protease genes of *Escherichia coli* K-12 from clustered Esigma32-specific promoters during heat shock. J. Bacteriol..

[31] Tsui H.C., Winkler M.E. (1994). Transcriptional patterns of the *mutL*-*miaA* superoperon of *Escherichia coli* K-12 suggest a model for posttranscriptional regulation. Biochimie.

[32] Tsui H.C., Feng G., Winkler M.E. (1997). Negative regulation of *mutS* and *mutH* repair gene expression by the *Hfq* and *RpoS* global regulators of *Escherichia coli* K-12. J. Bacteriol..

[33] Py B., Causton H., Mudd E.A., Higgins C.F. (1994). A protein complex mediating mRNA degradation in *Escherichia coli*. Mol. Microbiol..

[34] Py B., Higgins C.F., Krisch H.M., Carpousis A.J. (1996). A DEAD-box RNA helicase in the *Escherichia coli* RNA degradosome. Nature.

[35] Mudd E.A., Higgins C.F. (1993). *Escherichia coli* endoribonuclease RNase E: Autoregulation of expression and site-specific cleavage of mRNA. Mol. Microbiol..

[36] Masse E., Escorcia F.E., Gottesman S. (2003). Coupled degradation of a small regulatory RNA and its mRNA targets in *Escherichia coli*. Genes Dev..

[37] Guillier M., Gottesman S., Storz G. (2006). Modulating the outer membrane with small RNAs. Genes Dev..

[38] Mandin P., Gottesman S. (2010). Integrating anaerobic/aerobic sensing and the general stress response through the ArcZ small RNA. EMBO J..

[39] Luo X., Majdalani N. (2024). Directed Screening for sRNA Targets in *E. coli* Using a Plasmid Library. Methods Mol. Biol..

[40] Liu M.Y., Gui G., Wei B., Preston J.F., Oakford L., Yuksel U., Giedroc D.P., Romeo T. (1997). The RNA molecule CsrB binds to the global regulatory protein CsrA and antagonizes its activity in *Escherichia coli*. J. Biol. Chem..

[41] Liu M.Y., Romeo T. (1997). The global regulator CsrA of *Escherichia coli* is a specific mRNA-binding protein. J. Bacteriol..

[42] Sabnis N.A., Yang H., Romeo T. (1995). Pleiotropic regulation of central carbohydrate metabolism in *Escherichia coli* via the gene *csrA*. J. Biol. Chem..

[43] Pannuri A., Vakulskas C.A., Zere T., McGibbon L.C., Edwards A.N., Georgellis D., Babitzke P., Romeo T. (2016). Circuitry Linking the Catabolite Repression and Csr Global Regulatory Systems of *Escherichia coli*. J. Bacteriol..

[44] Wang X., Dubey A.K., Suzuki K., Baker C.S., Babitzke P., Romeo T. (2005). CsrA post-transcriptionally represses *pgaABCD*, responsible for synthesis of a biofilm polysaccharide adhesin of *Escherichia coli*. Mol. Microbiol..

[45] Wei B.L., Brun-Zinkernagel A.M., Simecka J.W., Pruss B.M., Babitzke P., Romeo T. (2001). Positive regulation of motility and *flhDC* expression by the RNA-binding protein CsrA of *Escherichia coli*. Mol. Microbiol..

[46] Wang D., McAteer S.P., Wawszczyk A.B., Russell C.D., Tahoun A., Elmi A., Cockroft S.L., Tollervey D., Granneman S., Tree J.J. (2018). An RNA-dependent mechanism for transient expression of bacterial translocation filaments. Nucleic. Acids Res..

[47] Liaw S.J., Lai H.C., Ho S.W., Luh K.T., Wang W.B. (2003). Role of RsmA in the regulation of swarming motility and virulence factor expression in *Proteus mirabilis*. J. Med. Microbiol..

[48] Nava-Galeana J., Yakhnin H., Babitzke P., Bustamante V.H. (2023). CsrA Positively and Directly Regulates the Expression of the *pdu*, *pocR*, and *eut* Genes Required for the Luminal Replication of *Salmonella Typhimurium*. Microbiol. Spectr..

[49] Zhu D., Wang S., Sun X. (2021). FliW and CsrA Govern Flagellin (FliC) Synthesis and Play Pleiotropic Roles in Virulence and Physiology of *Clostridioides difficile* R20291. Front Microbiol.

[50] Hubloher J.J., Schabacker K., Muller V., Averhoff B. (2021). CsrA Coordinates Compatible Solute Synthesis in *Acinetobacter baumannii* and Facilitates Growth in Human Urine. Microbiol Spectr.

[51] Butz H.A., Mey A.R., Ciosek A.L., Crofts A.A., Davies B.W., Payne S.M. (2021). Regulatory Effects of CsrA in *Vibrio cholerae*. mBio.

[52] Dai Q., Xu L., Xiao L., Zhu K., Song Y., Li C., Zhu L., Shen X., Wang Y. (2018). RovM and CsrA Negatively Regulate Urease Expression in *Yersinia pseudotuberculosis*. Front. Microbiol..

[53] Potts A.H., Leng Y., Babitzke P., Romeo T. (2018). Examination of Csr regulatory circuitry using epistasis analysis with RNA-seq (Epi-seq) confirms that CsrD affects gene expression via CsrA, CsrB and CsrC. Sci. Rep..

[54] Heroven A.K., Bohme K., Dersch P. (2012). The Csr/Rsm system of *Yersinia* and related pathogens: A post-transcriptional strategy for managing virulence. RNA Biol..

[55] Abbott Z.D., Yakhnin H., Babitzke P., Swanson M.S. (2015). *csrR*, a Paralog and Direct Target of CsrA, Promotes *Legionella pneumophila* Resilience in Water. mBio.

[56] Ozturk G., LeGrand K., Zheng Y., Young G.M. (2017). *Yersinia enterocolitica* CsrA regulates expression of the Ysa and Ysc type 3 secretion system in unique ways. FEMS Microbiol. Lett..

[57] Muller P., Gimpel M., Wildenhain T., Brantl S. (2019). A new role for CsrA: Promotion of complex formation between an sRNA and its mRNA target in *Bacillus subtilis*. RNA Biol..

[58] Heroven A.K., Nuss A.M., Dersch P. (2017). RNA-based mechanisms of virulence control in Enterobacteriaceae. RNA Biol..

[59] Vakulskas C.A., Potts A.H., Babitzke P., Ahmer B.M., Romeo T. (2015). Regulation of bacterial virulence by Csr (Rsm) systems. Microbiol. Mol. Biol. Rev. MMBR.

[60] Cerca N., Jefferson K.K. (2008). Effect of growth conditions on poly-N-acetylglucosamine expression and biofilm formation in *Escherichia coli*. FEMS Microbiol. Lett..

[61] Lee J.H., Ancona V., Chatnaparat T., Yang H., Zhao Y. (2019). The RNA-binding protein CsrA controls virulence in *Erwinia amylovora* by regulating RelA, RcsB, and FlhD at the posttranscriptional level. Mol. Plant Microbe Interact..

[62] Rojano-Nisimura A.M., Simmons T.R., Leistra A.N., Mihailovic M.K., Buchser R., Ekdahl A.M., Joseph I., Curtis N.C., Contreras L.M. (2023). CsrA selectively modulates sRNA-mRNA regulator outcomes. Front. Mol. Biosci..

[63] Stenum T.S., Holmqvist E. (2022). CsrA enters *Hfq*’s territory: Regulation of a base-pairing small RNA. Mol. Microbiol..

[64] Lai Y.J., Yakhnin H., Pannuri A., Pourciau C., Babitzke P., Romeo T. (2022). CsrA regulation via binding to the base-pairing small RNA Spot 42. Mol. Microbiol..

[65] London L.Y., Aubee J.I., Nurse J., Thompson K.M. (2021). Post-transcriptional regulation of rseA by small RNAs *ryhB* and *fnrS* in *Escherichia coli*. Front. Mol. Biosci..

[66] Jorgensen M.G., Thomason M.K., Havelund J., Valentin-Hansen P., Storz G. (2013). Dual function of the McaS small RNA in controlling biofilm formation. Genes Dev..

[67] Rojano-Nisimura A.M., Simmons T.R., Lukasiewicz A.J., Buchser R., Ruzek J.S., Avila J.L., Contreras L.M. (2025). Concentration-Dependent CsrA Regulation of the *uxuB* Transcript Leads to Development of a Post-Transcriptional Bandpass Filter. ACS Synth. Biol..

[68] Rojano-Nisimura A.M., Grismore K.B., Ruzek J.S., Avila J.L., Contreras L.M. (2024). The Post-Transcriptional Regulatory Protein CsrA Amplifies Its Targetome through Direct Interactions with Stress-Response Regulatory Hubs: The EvgA and AcnA Cases. Microorganisms.

[69] Leistra A.N., Gelderman G., Sowa S.W., Moon-Walker A., Salis H.M., Contreras L.M. (2018). A Canonical Biophysical Model of the CsrA Global Regulator Suggests Flexible Regulator-Target Interactions. Sci. Rep..

[70] Romeo T. (1998). Global regulation by the small RNA-binding protein CsrA and the non-coding RNA molecule CsrB. Mol. Microbiol..

[71] Romeo T., Babitzke P. (2018). Global Regulation by CsrA and Its RNA Antagonists. Microbiol. Spectr..

[72] Kudla G., Murray A.W., Tollervey D., Plotkin J.B. (2009). Coding-sequence determinants of gene expression in *Escherichia coli*. Science.

[73] El Yacoubi B., Bailly M., de Crecy-Lagard V. (2012). Biosynthesis and function of posttranscriptional modifications of transfer RNAs. Annu. Rev. Genet..

[74] de Crecy-Lagard V., Jaroch M. (2021). Functions of Bacterial tRNA Modifications: From Ubiquity to Diversity. Trends Microbiol..

[75] de Crecy-Lagard V., Ross R.L., Jaroch M., Marchand V., Eisenhart C., Bregeon D., Motorin Y., Limbach P.A. (2020). Survey and Validation of tRNA Modifications and Their Corresponding Genes in *Bacillus subtilis* sp. Subtilis Strain 168. Biomolecules.

[76] Quaiyum S., Sun J., Marchand V., Sun G., Reed C.J., Motorin Y., Dedon P.C., Minnick M.F., de Crecy-Lagard V. (2024). Mapping the tRNA modification landscape of *Bartonella henselae* Houston I and *Bartonella quintana* Toulouse. Front. Microbiol..

[77] McGuffey J.C., Jackson-Litteken C.D., Di Venanzio G., Zimmer A.A., Lewis J.M., Distel J.S., Kim K.Q., Zaher H.S., Alfonzo J., Scott N.E. (2023). The tRNA methyltransferase TrmB is critical for *Acinetobacter baumannii* stress responses and pulmonary infection. mBio.

[78] Diaz-Rullo J., Gonzalez-Pastor J.E. (2023). tRNA queuosine modification is involved in biofilm formation and virulence in bacteria. Nucleic Acids Res..

[79] Shippy D.C., Eakley N.M., Lauhon C.T., Bochsler P.N., Fadl A.A. (2013). Virulence characteristics of *Salmonella* following deletion of genes encoding the tRNA modification enzymes GidA and MnmE. Microb. Pathog..

[80] Krueger J., Preusse M., Oswaldo Gomez N., Frommeyer Y.N., Doberenz S., Lorenz A., Kordes A., Grobe S., Musken M., Depledge D.P. (2024). tRNA epitranscriptome determines pathogenicity of the opportunistic pathogen *Pseudomonas aeruginosa*. Proc. Natl. Acad. Sci. USA.

[81] Zhong W., Koay A., Ngo A., Li Y., Nah Q., Wong Y.H., Chionh Y.H., Ng H.Q., Koh-Stenta X., Poulsen A. (2019). Targeting the Bacterial Epitranscriptome for Antibiotic Development: Discovery of Novel tRNA-(N(1)G37) Methyltransferase (TrmD) Inhibitors. ACS Infect. Dis..

[82] Zhong W., Pasunooti K.K., Balamkundu S., Wong Y.H., Nah Q., Gadi V., Gnanakalai S., Chionh Y.H., McBee M.E., Gopal P. (2019). Thienopyrimidinone Derivatives That Inhibit Bacterial tRNA (Guanine37-N(1))-Methyltransferase (TrmD) by Restructuring the Active Site with a Tyrosine-Flipping Mechanism. J. Med. Chem..

[83] Hofer K., Jaschke A. (2018). Epitranscriptomics: RNA Modifications in Bacteria and Archaea. Microbiol. Spectr..

[84] Zhao B.S., Roundtree I.A., He C. (2017). Post-transcriptional gene regulation by mRNA modifications. Nat. Rev. Mol. Cell Biol..

[85] Luo H., Wei J., Wu S., Zheng Q., Zhang N., Chen P. (2023). Exploring CircRNA N6-methyladenosine in human rheumatoid arthritis: Hyper-methylated hsa_circ_0007259 as a potential biomarker and its involvement in the hsa_circ_0007259/hsa_miR-21-5p/STAT3 axis. Int. Immunopharmacol..

[86] He T., Hia H., Chen B., Duan Z., Huang C. (2023). m6A Writer mettl3-mediated lncRNA linc01125 prevents the malignancy of papillary thyroid cancer. Crit. Rev. Immunol..

[87] Ma L., Ma Q., Deng Q., Zhou J., Zhou Y., Wei Q., Huang Z., Lao X., Du P. (2023). N7-methylguanosine-related miRNAs predict hepatocellular carcinoma prognosis and immune therapy. Aging.

[88] Chan C.T., Deng W., Li F., DeMott M.S., Babu I.R., Begley T.J., Dedon P.C. (2015). Highly Predictive Reprogramming of tRNA Modifications Is Linked to Selective Expression of Codon-Biased Genes. Chem. Res. Toxicol..

[89] Deng W., Babu I.R., Su D., Yin S., Begley T.J., Dedon P.C. (2015). Trm9-Catalyzed tRNA Modifications Regulate Global Protein Expression by Codon-Biased Translation. PLoS Genet..

[90] de Crecy-Lagard V., Boccaletto P., Mangleburg C.G., Sharma P., Lowe T.M., Leidel S.A., Bujnicki J.M. (2019). Matching tRNA modifications in humans to their known and predicted enzymes. Nucleic. Acids. Res..

[91] Helm M., Alfonzo J.D. (2014). Posttranscriptional RNA Modifications: Playing Metabolic Games in a Cell’s Chemical Legoland. Chem. Biol..

[92] Gu C., Begley T.J., Dedon P.C. (2014). tRNA modifications regulate translation during cellular stress. FEBS Lett..

[93] Dedon P.C., Begley T.J. (2014). A system of RNA modifications and biased codon use controls cellular stress response at the level of translation. Chem. Res. Toxicol..

[94] Pollo-Oliveira L., Davis N.K., Hossain I., Ho P., Yuan Y., Salguero Garcia P., Pereira C., Byrne S.R., Leng J., Sze M. (2022). The absence of the queuosine tRNA modification leads to pleiotropic phenotypes revealing perturbations of metal and oxidative stress homeostasis in *Escherichia coli* K12. Metallomics.

[95] Chionh Y.H., McBee M., Babu I.R., Hia F., Lin W., Zhao W., Cao J., Dziergowska A., Malkiewicz A., Begley T.J. (2016). tRNA-mediated codon-biased translation in mycobacterial hypoxic persistence. Nat. Commun..

[96] Lampi M., Gregorova P., Qasim M.S., AAhlblad N.C.V., Sarin L.P. (2023). Bacteriophage infection of the marine bacterium *shewanella glacialimarina* induces dynamic change in tRNA modifications. Microorganisms.

[97] Nakayashiki T., Inokuchi H. (1998). Novel temperature-sensitive mutants of *Escherichia coli* that are unable to grow in the absence of wild type tRNA_6_^Leu^. J. Bacteriol..

[98] Renda A., Poly S., Lai Y.J., Pannuri A., Yakhnin H., Potts A.H., Bevilacqua P.C., Romeo T., Babitzke P. (2020). CsrA-Mediated Translational Activation of ymdA Expression in *Escherichia coli*. mBio.

[99] Vakulskas C.A., Leng Y., Abe H., Amaki T., Okayama A., Babitzke P., Suzuki K., Romeo T. (2016). Antagonistic control of the turnover pathway for the global regulatory sRNA CsrB by the CsrA and CsrD proteins. Nucleic. Acids Res..

[100] Kitagawa R., Mitsuki H., Okazaki T., Ogawa T. (1996). A novel DnaA protein-binding site at 94.7 min on the *Escherichia coli* chromosome. Mol. Microbiol..

[101] Maciag A., Peano C., Pietrelli A., Egli T., De Bellis G., Landini P. (2011). In vitro transcription profiling of the sigmaS subunit of bacterial RNA polymerase: Re-definition of the sigmaS regulon and identification of sigmaS-specific promoter sequence elements. Nucleic. Acids Res..

[102] Tsui H.C., Leung H.C., Winkler M.E. (1994). Characterization of broadly pleiotropic phenotypes caused by an *hfq* insertion mutation in *Escherichia coli* K-12. Mol. Microbiol..

[103] Brown L., Elliot T. (1996). Efficient translation of the *RpoS* sigma factor in *Salmonella typhimurium* requires host factor I, an RNA-binding protein encoded by the *hfq* gene. J. Bacteriol..

[104] Brown L., Elliot T. (1997). Mutations that increase expression of the *rpoS* gene and decrease its dependence on *hfq* function in *Salmonella typhimurium*. J. Bacteriol..

[105] Zhang A., Altuvia S., Tiwari A., Argaman L., Hengge-Aronis R., Storz G. (1998). The OxyS regulatory RNA represses *rpoS* translation and binds the Hfq (HF-I) protein. EMBO J..

[106] Sledjeski D.D., Whitman C., Zhang A. (2001). Hfq is necessary for regulation by the untranslated RNA DsrA. J. Bacteriol..

[107] Majdalani N., Chen S., Murrow J., St John K., Gottesman S. (2001). Regulation of *RpoS* by a novel small RNA: The characterization of RprA. Mol. Microbiol..

[108] Baker C.S., Eory L.A., Yakhnin H., Mercante J., Romeo T., Babitzke P. (2007). CsrA inhibits translation initiation of *Escherichia coli hfq* by binding to a single site overlapping the shine-dalgarno aequence. J. Bacteriol..

[109] Yu D., Ellis H.M., Lee E.C., Jenkins N.A., Copeland N.G., Court D.L. (2000). An efficient recombination system for chromosome engineering in *Escherichia coli*. Proc. Natl. Acad. Sci. USA.

[110] Court D.L., Swaminathan S., Yu D., Wilson H., Baker T., Bubunenko M., Sawitzke J., Sharan S.K. (2003). Mini-lambda: A tractable system for chromosome and BAC engineering. Gene.

[111] Chung C.T., Niemela S.L., Miller R.H. (1989). One-step preparation of competent *Escherichia coli*: Transformation and storage of bacterial cells in the same solutions. Proc. Natl. Acad. Sci. USA.

[112] Sharan S.K., Thomason L.C., Kuznetsov S.G., Court D.L. (2009). Recombineering: A homologous recombination-based method of genetic engineering. Nat. Protoc..

[113] Thomason L.C., Costantino N., Court D.L. (2007). *E. coli* genome manipulation by P1 transduction. Curr. Protoc. Mol. Biol..

[114] Karlsson J., Eichner H., Loh E. (2023). Total Bacterial RNA Isolation and Northern Blotting Analysis. Methods Mol. Biol..

[115] Ares M. (2012). Bacterial RNA isolation. Cold Spring Harb. Protoc..

[116] Masse E., Gottesman S. (2002). A small RNA regulates the expression of genes involved in iron metabolism in *Escherichia coli*. Proc. Natl. Acad. Sci. USA.

[117] Rio D.C. (2015). Northern blots: Capillary transfer of RNA from agarose gels and filter hybridization using standard stringency conditions. Cold Spring Harb. Protoc..

[118] Thibodeau S.A., Fang R., Joung J.K. (2004). High-throughput beta-galactosidase assay for bacterial cell-based reporter systems. BioTechniques.

